# Incorporation of Data From Multiple Hypervariable Regions when Analyzing Bacterial 16S rRNA Gene Sequencing Data

**DOI:** 10.3389/fgene.2022.799615

**Published:** 2022-03-31

**Authors:** Carli B. Jones, James R. White, Sarah E. Ernst, Karen S. Sfanos, Lauren B. Peiffer

**Affiliations:** ^1^ Department of Pathology, Johns Hopkins University School of Medicine, Baltimore, MD, United States; ^2^ Resphera Biosciences, Baltimore, MD, United States; ^3^ Deparment of Oncology, Johns Hopkins University School of Medicine, Baltimore, MD, United States; ^4^ Department of Urology, Johns Hopkins University School of Medicine, Baltimore, MD, United States; ^5^ Department of Molecular and Comparative Pathobiology, Johns Hopkins University School of Medicine, Baltimore, MD, United States

**Keywords:** 16S rRNA, microbiome, hypervariable regions, sequencing, ion torrent

## Abstract

Short read 16 S rRNA amplicon sequencing is a common technique used in microbiome research. However, inaccuracies in estimated bacterial community composition can occur due to amplification bias of the targeted hypervariable region. A potential solution is to sequence and assess multiple hypervariable regions in tandem, yet there is currently no consensus as to the appropriate method for analyzing this data. Additionally, there are many sequence analysis resources for data produced from the Illumina platform, but fewer open-source options available for data from the Ion Torrent platform. Herein, we present an analysis pipeline using open-source analysis platforms that integrates data from multiple hypervariable regions and is compatible with data produced from the Ion Torrent platform. We used the ThermoFisher Ion 16 S Metagenomics Kit and a mock community of twenty bacterial strains to assess taxonomic classification of six amplicons from separate hypervariable regions (V2, V3, V4, V6-7, V8, V9) using our analysis pipeline. We report that different amplicons have different specificities for taxonomic classification, which also has implications for global level analyses such as alpha and beta diversity. Finally, we utilize a generalized linear modeling approach to statistically integrate the results from multiple hypervariable regions and apply this methodology to data from a representative clinical cohort. We conclude that examining sequencing results across multiple hypervariable regions provides more taxonomic information than sequencing across a single region. The data across multiple hypervariable regions can be combined using generalized linear models to enhance the statistical evaluation of overall differences in community structure and relatedness among sample groups.

## Introduction

Next generation sequencing of microbial DNA has become an important tool used for determining relationships between human-associated microbial populations and various diseases. Most studies in this realm rely on either shotgun metagenomic sequencing or 16 S ribosomal RNA (rRNA) amplicon sequencing. Shotgun metagenomic sequencing involves sequencing random fragments of sample DNA which contains a mixture of bacterial DNA, as well as host and other microbial and environmental DNA (Quince et al., 2017). This method allows for taxonomic profiling, metabolic function profiling, and antibiotic resistance gene profiling; however, it is generally more expensive than amplicon sequencing, and requires a larger amount of input DNA and the availability of reference genome sequences. Bacterial 16 S rRNA amplicon sequencing employs PCR amplification of specific hypervariable regions within the gene, followed by deep sequencing (Sanschagrin and Yergeau, 2014). This method is generally a quicker, cheaper alternative to shotgun metagenomics; however, it only identifies bacteria and the typical strategy only sequences a specific fragment of the bacterial 16 S rRNA gene (Ranjan et al., 2016). While functional information can be inferred from taxonomic classification using tools such as UniRef and KEGG Orthology, the genetic elements contributing to these functions themselves are not sequenced. The 16 S rRNA gene is comprised of 9 hypervariable regions (V1-V9), and most primers used for next generation sequencing only target one to two hypervariable regions at a time. Multiple studies have shown that different regions vary in their taxonomic utility due to a combination of primer bias, differential hypervariable region sequence length, and hypervariable region sequence uniqueness across bacterial taxa (Claesson et al., 2010; Pinto and Raskin, 2012; Cai et al., 2013; Tremblay et al., 2015; Barb et al., 2016). An ideal solution would be to sequence the entire 16 S rRNA gene, however this technique is more costly and access to this technology is limited compared to traditional 16 S rRNA sequencing. Therefore, a potential alternative would be to perform 16 S rRNA amplicon sequencing on multiple regions and incorporate information from as many hypervariable regions as possible into downstream data analysis.

The Ion 16 S™ Metagenomics Kit (Life Technologies) utilizes six sets of primers spanning seven different hypervariable regions: V2, V3, V4, V6-7, V8, and V9. This is an attractive approach because it yields more sequence information across the 16 S rRNA gene overall. However, there is currently little consensus as to how to properly analyze information from multiple hypervariable regions and obtain overall results. Current analysis pipelines for Ion Torrent data include the Ion Reporter Software offered by ThermoFisher, and an alternative method using open access tools developed by Barb *et al.* (Barb et al., 2016). The utility of Ion Reporter Software is limited; for example, users are unable to incorporate study-specific metadata into analyses, and exported processed data is devoid of previous analysis information, preventing downstream analysis with open-source tools. Barb *et al.* offer methods for taxonomic identification; however, they do not address the question of how to appropriately integrate data from multiple hypervariable regions in downstream analyses. Recently, Fuks *et al.* (Fuks et al., 2018) and Debelius *et al.* (Debelius et al., 2021) developed methods to computationally combine data from multiple hypervariable regions to provide a joint estimate of the microbial community composition. To date, however, there is no generally agreed upon approach for combining sequences from multiple hypervariable regions for downstream analyses, especially for less commonly used 16 S rRNA gene sequencing platforms such as Ion Torrent.

Herein, we developed an analysis pipeline that analyzes data from each hypervariable region separately, allowing for systematic comparison of taxonomic classification by hypervariable region. We demonstrate our results from analyzing a mock community of bacterial DNA where we determine how each hypervariable region differs in its utility to provide information on taxonomic classifications, alpha diversity, and beta diversity. We report that certain taxa are only identified by particular hypervariable regions, corroborating prior studies (Claesson et al., 2010; Pinto and Raskin, 2012; Cai et al., 2013; Tremblay et al., 2015; Barb et al., 2016) and supporting our hypothesis that there is a benefit to incorporating multiple primer sets into sequencing strategies. Furthermore, we discuss different options for downstream analysis and statistics, and demonstrate that using a generalized linear model (GLM) to statistically combine results from multiple hypervariable regions increases sensitivity of taxonomic classification. Finally, we demonstrate the utility of our approach in the analysis of clinical samples in an illustrative clinical cohort.

## Materials and Methods

### Mock Community

The 20 Strain Even Mix Genomic Material was obtained from American Type Culture Collection (ATCC, Cat. No. MSA-1002, Manassas, VA). The strain composition of the mock community is given in Table 1. The mock community was sequenced a total of five times from four library preparations and over three sequencing runs.

**TABLE 1:** Contents of mock community.

Species	16S copies^a^	Genus	Family
*Acinetobacter baumannii*	6	*Acinetobacter*	*Moraxellaceae*
*Actinomyces odontolyticus*	2	*Actinomyces*	*Actinomycetaceae*
*Bacillus cereus*	12	*Bacillus*	*Bacillaceae*
*Bacteroides vulgatus*	7	*Bacteroides*	*Bacteroidaceae*
*Bifidobacterium adolescentis*	5	*Bifidobacterium*	*Bifidobacteriaceae*
*Clostridium beijerinckii*	14	*Clostridium*	*Clostridiaceae*
*Cutibacterium acnes*	4	*Cutibacterium*	*Propionibacteriaceae*
*Deinococcus radiodurans*	7	*Deinococcus*	*Deinococcaceae*
*Enterococcus faecalis*	4	*Enterococcus*	*Enterococcaceae*
*Escherichia coli*	7	*Escherichia*	*Enterobacteriaceae*
*Helicobacter pylori*	2	*Helicobacter*	*Helicobacteraceae*
*Lactobacillus gasseri*	6	*Lactobacillus*	*Lactobacillaceae*
*Neisseria meningitidis*	4	*Neisseria*	*Neisseriaceae*
*Porphyromonas gingivalis*	4	*Porphorymonas*	*Porphyromonadaceae*
*Pseudomonas aeruginosa*	4	*Pseudomonas*	*Pseudomonadaceae*
*Rhodobacter sphaeroides*	3	*Rhodobacter*	*Rhodobacteraceae*
*Staphylococcus aureus*	6	*Staphylococcus*	*Staphylococcaceae*
*Staphylococcus epidermidis*	5	*Staphylococcus*	*Staphylococcaceae*
*Streptococcus agalactiae*	7	*Streptococcus*	*Streptococcaceae*
*Streptococcus mutans*	5	*Streptococcus*	*Streptococcaceae*

a Number of copies of 16S rRNA genes contained in the bacterial genome of the indicated species.

### Clinical Sample Collection

All specimens were studied under an Institutional Review Board (IRB) approved protocol with written informed consent. A total of three (3) adult males self-collected two (2) rectal swab samples each with sterile flocked swabs (Cat. No. 552C, Copan Diagnostics, Murrieta, CA). One rectal swab from each individual was randomly selected for DNA extraction immediately after sample collection (RS1). The other swab (RS2) was frozen at –80°C for 6 days before DNA extraction.

### DNA Extraction

The DNA extraction protocol was adapted from our previously published protocol (Shrestha et al., 2018). Briefly, rectal swab fecal material was resuspended in 500 µl of 1X phosphate buffered saline (PBS) (Cat. No. 21-031-CV, Corning, Manassas, VA). Samples were then digested in a cocktail of lysozyme (10 mg/ml, Cat. No. L7773, Sigma-Aldrich, St. Louis, MO) and mutanolysin (25 KU/ml, Cat. No. M4782, Sigma-Aldrich, St. Louis, MO) for 1 h at 37°C. The contents of the tubes were then transferred into FastPrep Lysing Matrix B tubes (Cat. No. 6911050, MP Biomedicals, Santa Ana, CA). Next, 20% SDS (Cat. No. 05030, Sigma-Aldrich, St. Louis, MO) and phenol:chloroform:isoamyl alcohol (25:24:1, Cat. No. 108-95-2, ThermoFisher Scientific, Waltham, MA) were added and samples were homogenized by bead beating in an MP FastPrep-24 at 6 m/s for a total of 60 s. DNA was precipitated and resuspended in a final volume of 50 μl of DNA-free water (Cat. No. P-020-0003, Molzym, Bremen, Germany).

### Library Preparation

Concentration of DNA from the mock microbial community (Table 1) and rectal swabs was measured using a Qubit dsDNA HS (high sensitivity) kit (Cat. No. Q32851, Life Technologies, Carlsbad, CA). Libraries were prepared using the Ion 16 S™ Metagenomics Kit (Cat. No. A26216, ThermoFisher Scientific, Waltham, MA). Briefly, 10 ng of DNA was mixed with 15 µl of Environmental Master Mix. 3 µl of each 16 S Primer Set (10X) was added to each tube, one sample set with primers for V2-4-8 (Pool 1) and the other with primers for V3-6,7-9 (Pool 2). Samples were placed in a thermocycler with the following thermal conditions: 95°C for 10 min; then 25 cycles of 95°C for 30 s, 58°C for 30s, 72°C for 30 s; and finally 72°C for 7 min. Amplification products were purified using AMPure XP beads (Cat. No. A63881, Beckman Coulter, Pasadena, CA) and eluted in nuclease free water. Concentrations of amplification products from Pool 1 and Pool 2 were measured using a Bioanalyzer High Sensitivity DNA Kit (Cat. No. 5067-4626, Agilent Technologies, Santa Clara, CA), and the two pools were combined for a total of 100 ng of DNA (50 ng from each pool).

Next, 20 µl of 5X End Repair Buffer and 1 µl of End Repair Enzyme were added to each sample, and then incubated for 20 min at room temperature. Pooled amplicons were then purified again using AMPure XP beads and eluted in Low TE buffer. Ligation and nick repair were performed using ×10 Ligase Buffer, Ion P1 Adaptor, Ion Xpress Barcodes, dNTP Mix, DNA Ligase, Nick Repair Polymerase, nuclease-free water, and sample DNA with the following thermal conditions: 25°C for 15 min, 72°C for 5 min. Adapter-ligated and nick-repaired DNA was then purified using AMPure XP beads and eluted in Low TE buffer.

The library was then amplified using the Ion Plus Fragment Library Kit (Cat. No. 4471252, ThermoFisher Scientific) with the following thermal conditions: 95°C for 5 min; then 7 cycles of 95°C for 15 s, 58°C for 15 s, 70°C for 1 min; and then finally 70°C for 1 min. The amplified library was then purified using AMPure XP beads and eluted in Low TE buffer. Library concentrations were measured using a Bioanalyzer and the High Sensitivity DNA Kit. Libraries were then diluted down to 26 pM and pooled, yielding a 26 pM solution.

### Sequencing

Libraries were prepared for sequencing using oil amplification to template the libraries onto beads and loaded onto chips using the Ion Chef Instrument and the Ion 520™ & Ion 530™ Kit–Chef (ThermoFisher Scientific). Chips were then loaded onto the Ion GeneStudio S5 System along with Ion S5 Sequencing Kit reagents (Cat. No. A35850, ThermoFisher Scientific, Waltham, MA) and sequenced at the Sidney Kimmel Comprehensive Cancer Center Experimental and Computational Genomics Core facility. Samples in this study were sequenced across three separate sequencing runs on Ion 520 and Ion 530 chips using 400bp sequencing kits. Sequences were demultiplexed by sample using the S5 device software, and then separated per hypervariable region by ThermoFisher prior to downstream analysis.

### Data Processing

Primer sequences are not made available to Ion 16 S™ Metagenomics Kit users. Therefore, FASTQ files had to be separated by primer set by the ThermoFisher Bioinformatics team, resulting in six separate FASTQ files per sample (V2, V3, V4, V6-7, V8, and V9), with primer sequences removed and all reads oriented in the forward direction.

Manifest files were then created for each hypervariable region and each sequencing run. FASTQ files were imported into QIIME2 format *via* qiime tools import in SingleEndFastqManifestPhred33V2 format (Bolyen et al., 2019). QIIME2 v 2020.6 was used to perform denoising, Operational Taxonomic Unit (OTU) clustering, taxonomic classification, phylogenetic tree construction, and alpha and beta diversity.

DADA2 was used to denoise data, using the denoise-pyro plugin and parameters of 0 bp for trimming and truncation (Callahan et al., 2016). A separate DADA2 run was performed for each hypervariable region and each sequencing run. Denoising statistics were then summarized and exported to P03-summarize-qc and P13-summarize-qc directories in the analysis folder of the it-workflow repository for the ATCC mock community samples and the clinical samples, respectively. From these summaries, we determined that all samples in all hypervariable regions had a minimum of 10,000 reads which passed the filter in the DADA2 step. Good’s coverage was performed at a depth of 10,000 reads for each hypervariable region and at least 99% coverage was achieved for all regions (Good, 1953). Thus, we decided that 10,000 reads was an acceptable sampling depth. DADA2 feature tables and representative sequence files were then merged across sequencing runs so that there was only one feature table and representative sequence file per hypervariable region.

Open-reference OTU clustering was then performed using QIIME2 plugin vsearch cluster-features-open-reference (Bokulich et al., 2018). A threshold of 99% identity was used, and sequences were clustered against reference sequences from the curated sfanos_db_v4.0 database as described below.

### Alpha and Beta Diversity Analysis

A phylogenetic tree was constructed for each hypervariable region using the “representative sequences” file generated from open-reference OTU clustering *via* the QIIME phylogeny align-to-tree-mafft-fasttree plugin (Faith et al., 1987; Price et al., 2010; Katoh and Standley, 2013). Community diversity was analyzed using the core-metrics-phylogenetic plugin. Briefly, the feature table produced by open-reference OTU clustering and the phylogenetic trees constructed in the previous step were input into the core-metrics-phylogenetic plugin, which performed alpha and beta diversity analyses at a sampling depth of 10,000 reads. Alpha diversity summaries were obtained and exported for Faith’s phylogenetic diversity, Shannon diversity (Shannon, 2001), evenness, and observed OTUs. Distance matrices were exported for Jaccard (Jaccard, 1908), Bray-Curtis (Sorensen, 1948), weighted UniFrac (Lozupone et al., 2007), and unweighted UniFrac (Lozupone and Knight, 2005) distances. Data was imported into Rstudio for visualization of alpha diversity metrics and principal coordinates analysis (PCoA). Taxonomic classification results from each hypervariable region were aggregated into summary tables at higher taxonomic levels (phylum through species) for downstream comparative analysis. Beta-diversity distance matrices (using the measures bray-curtis, jaccard, unweighted-unifrac, and weighted-unifrac) were based on OTU profiles and were generated for each hypervariable region separately to account for region-specific OTUs. Additionally, a multi-region beta-diversity analysis incorporated species level assignments across all hypervariable regions, followed by distance matrix calculation (Canberra, Bray-Curtis, Jaccard, Euclidean, Gower, and Kulczynski) using the vegdist command in the vegan R package.

### Database Curation

It is well known that curating existing taxonomic databases can lead to improved performance (Ritari et al., 2015; Clemmons et al., 2019; Myer et al., 2020). Therefore, uncultured and unclassified sequences were removed from the SILVA (v.123) database to eliminate sequences that have no practical value in taxonomic assignment. This refined database (*sfanos-db-4.0*) contains approximately 15,000 named species.

### *In Silico* Taxonomic Validation of Curated Database

Prior to using sfanos-db-4.0 for taxonomic classification, we verified its utility by performing *in silico* taxonomic classification using sequences from a published human gut microbiome culture collection (Forster et al., 2019). First, we separated the sequences in the culture collection by hypervariable region to mimic our own data. To do this, we ran the sequences from the culture collection through NCBI BLAST against the ATCC mock community sequences that had already been split by hypervariable region. This method allowed us to break down the culture collection sequences into their different hypervariable regions and simulate more complex clinical data. A 1% noise rate was included in the simulated sequences to mimic typical evolutionary variation in species as well as sequencing error. We then ran taxonomic classification of the sequences from the culture collection using our curated database, with a threshold of 97% sequence identity. A confidence score was assigned to each classification by VSEARCH. Results were categorized into true positives (TP), false positives (FP), and false negatives (FN) based on whether they were found in the culture collection or not (Supplementary File S1). Sequence assignment counts were converted to percent by adding up the total number of sequences that were assigned as TP, FP, or FN for each V region, dividing by the total number of sequences for that region, and multiplying by 100.

### Taxonomic Classification

Taxonomic classification was performed using classify-consensus-vsearch using the curated sfanos_db_v4.0 reference reads and reference taxonomy with 99% identity. The output. qza file was then exported in order to obtain the taxonomy. tsv file. This file and the feature-table. biom file were used in a Perl script designed to summarize the taxonomic information into feature-table-with-taxonomy.txt. Heatmaps were created in R using the pheatmap package and taxa-normalize-pct-per-region.txt file.

### Contaminant Filtering

Contaminant sequences were filtered out from the ATCC sample data. Any taxa that were detected in only one of the five technical replicates, detected at less than 0.1% abundance, or both, was considered a contaminant. Filtering was performed on the feature table that was created after open reference OTU clustering using QIIME taxa filter-table. Contaminants are listed in Supplementary Table S1.

### Generalized Linear Modeling

We used the generalized linear model function in Base R to evaluate statistical differences in alpha diversity and individual taxonomic abundance between fresh versus frozen samples in the clinical cohort. The GLM per feature took the following structure: log*10(feature) ∼ fresh/frozen status + specimen ID + hypervariable region.* Regions V8 and V9 were excluded from GLM analysis, and Region V2 was used as the null factor level. The fresh/frozen status of samples was compared, with fresh as baseline factor level set as zero and frozen set as one. The input of “feature” was either an alpha diversity value (Shannon, evenness, observed OTUs or Faith’s phylogenetic diversity), or taxonomic abundance of a feature at a specific taxonomic level. Input feature values were log transformed in order to increase stability of values from person to person when performing statistics. The GLM p-value was obtained by comparing the GLM factor level coefficient to the null hypothesis of zero, which was done *via* a Wald Test.

### Data and Code Availability

All sequence files are available in the NCBI Sequence Read Archive (SRA) under Bioproject ID PRJNA738491 (https://www.ncbi.nlm.nih.gov/bioproject/PRJNA738491). All codes are available on the public GitHub repository it-workflow (http://github.com/Sfanos-Lab-Microbiome-Projects/it-workflow/).

## Results

### Mock Community

**FIGURE 1:**
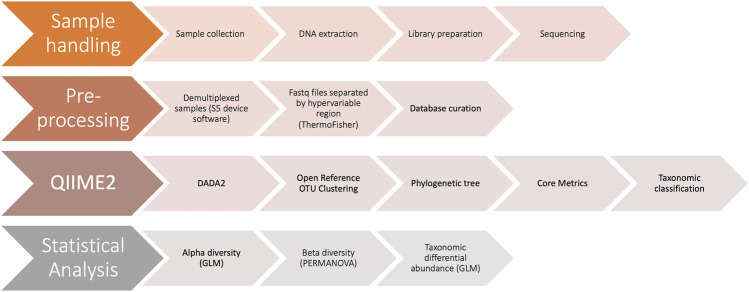
Schematic diagram of workflow. The four major steps in our workflow include 1) sample handling, from sample collection to sequencing 2) pre-processing of sequencing data and taxonomic reference database 3) performing microbiome bioinformatics using QIIME2 and 4) statistical analysis of results using R.

In order to test our analysis pipeline (Figure 1) we prepared libraries and sequenced DNA from a mock microbial community (Table 1). A total of five independent replicates from four library preparations of the mock community were sequenced over three sequencing runs. We filtered out low-level contaminants (Supplementary Table S1) prior to performing community alpha and beta diversity and taxonomic abundance analyses (see Methods).

**FIGURE 2:**
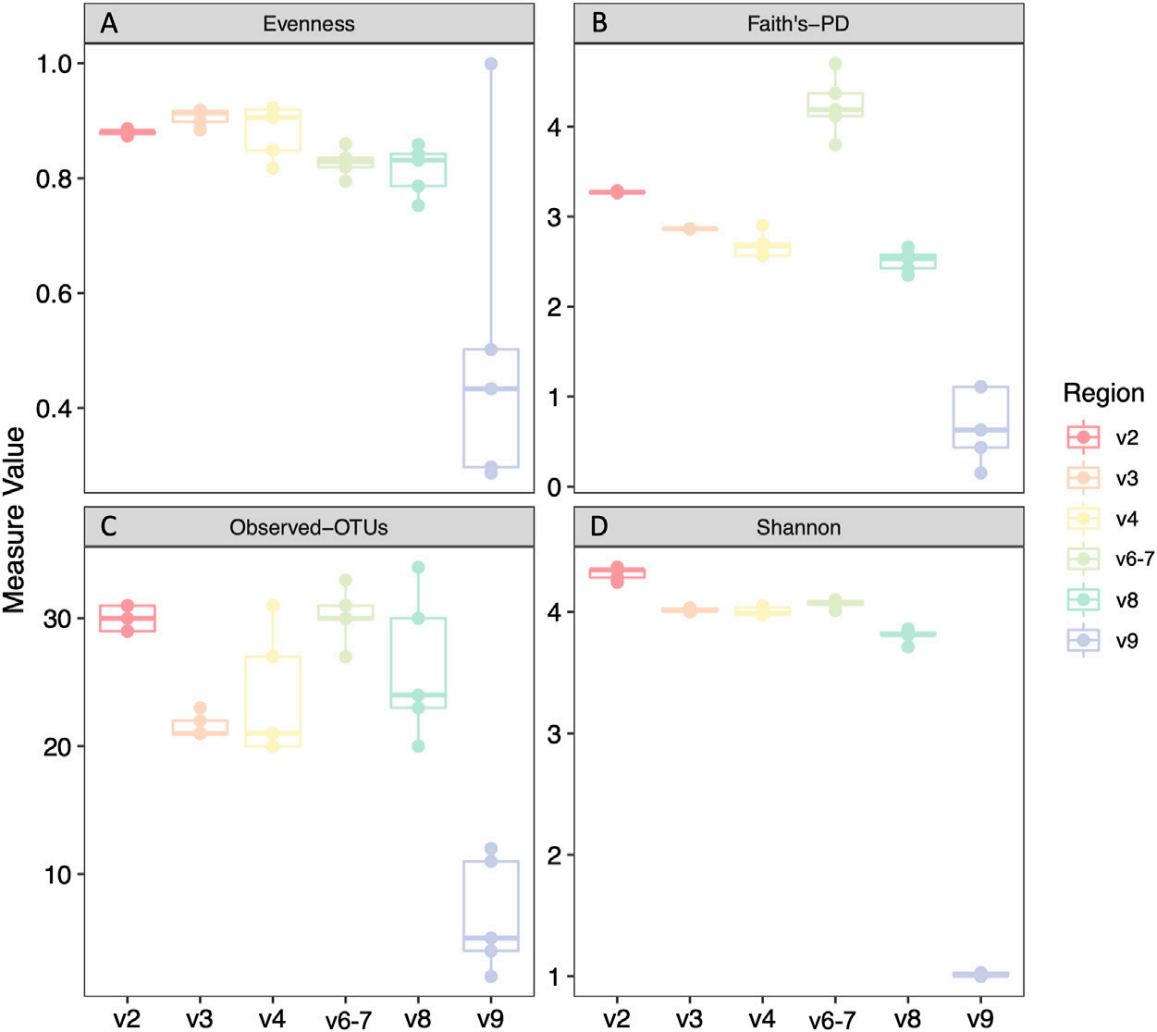
Alpha diversity analyses of mock community technical replicates by hypervariable region. Evenness **(A)**, Faith’s phylogenetic diversity **(B)**, Observed Operational Taxonomic Units (OTUs) **(C)**, Shannon diversity **(D)**. Statistical analysis and p values can be found in Supplementary File S2.

We analyzed four different alpha diversity metrics: two measures of evenness (evenness and Shannon diversity), and two measures of richness (Faith’s phylogenetic diversity and observed-OTUs) (Figure 2). V9 had significantly decreased alpha diversity compared to all regions across all metrics (Supplementary File S2). V8 also had significantly decreased Shannon diversity, evenness, and Faith’s phylogenetic diversity compared to other regions excluding V9, with two exceptions being that Evenness was not significantly decreased in V8 compared to that of V6-7 and Faith’s PD is not significantly decreased in V8 compared to V4 (Supplementary File S2).

**FIGURE 3:**
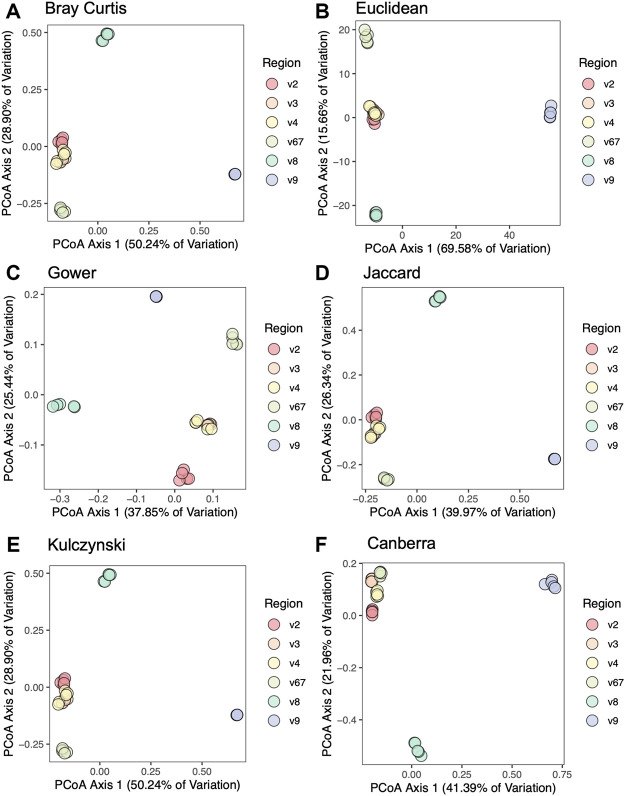
Principal coordinates analysis of mock community samples. PCoA plots are based on distance matrices for **(A)** Bray-Curtis, **(B)** Euclidean, **(C)** Gower, **(D)** Jaccard, **(E)** Kulczynski, and **(F)** Canberra.

To compare beta diversity between hypervariable regions and circumvent the issue that OTUs would be region-specific, we used taxonomic results from each hypervariable region to create aggregated distance matrices. We assessed six different beta diversity metrics: Canberra, Bray-Curtis, Jaccard, Euclidean, Gower, and Kulczynski. Figure 3 shows PCoA plots based on six different beta diversity metrics: Bray-Curtis (Figure 3A), Euclidean (Figure 3B), Gower (Figure 3C), Jaccard (Figure 3D), Kulczynski (Figure 3E), and Canberra (Figure 3F). In the plot based on the Canberra distance matrix (Figure 3F), the V2, V3, V4, and V6-7 hypervariable regions clustered together, whereas V8 and V9 were distantly separated. This pattern was also observed by the other beta diversity metrics, with V6-7 sometimes also segregating slightly from V2, V3, and V4 which were largely clustered together.

In addition to biodiversity measurements and beta diversity metrics, the percent abundance of the identified organisms after taxonomic classification was evaluated and is given in Supplementary File S3. The majority of species were identified by taxonomic classification of the sequences covering each hypervariable region, with the exception of V9 that only positively identified *Escherichia coli* and *Acinetobacter baumannii*. *Clostridium beijerinckii* was the most difficult organism to speciate and was only correctly classified in V6-7 amplicons. The results with hypervariable regions V2, V3, and V4 only identified *Clostridium beijerinckii* at the genus level, V8 mis-classified it as *Clostridium butyricum*, and V9 did not identify any Clostridial organisms (Supplementary File S3). Aside from *C. beijerinckii*, species misclassification varied by hypervariable region.

**FIGURE 4:**
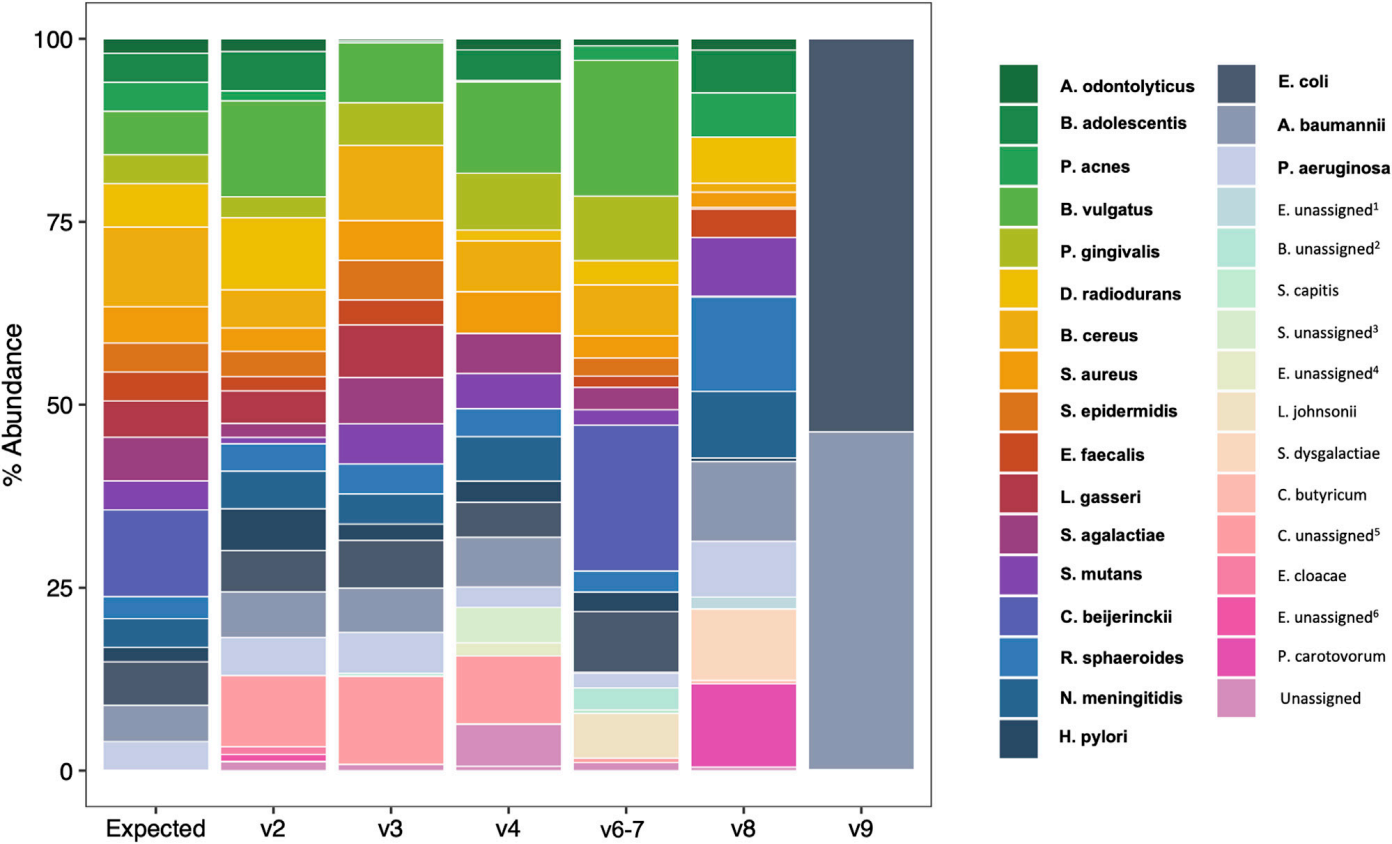
Species-level taxonomic barplots of ATCC 20 Strain Mix sequencing results by 16S rRNA hypervariable region. Bolded taxa are those present in the 20 strain mix. *Enterobacteriaceae* unassigned^1^, *Bifidobacterium* unassigned^2^, *Staphylococcus* unassigned^3^, *Enterococcus* unassigned^4^, *Clostridium sensu stricto 1* unassigned^5^, *Enterobacter* unassigned^6^.

We next compared observed versus expected percent abundance by hypervariable region. There are 114 copies of the 16 S rRNA gene in the bacterial genomes comprising the mock community. Therefore, the expected abundance of a given species’ rRNA gene is the number of copies in its genome (Table 1), divided by 114. Taxonomic bar plots demonstrate the percent abundance of each taxon by hypervariable region compared to expected (Figure 4). V2 most closely approximated the overall distribution of species compared to expected and correctly assigned the most species from the mock community (19/20). V3 (17/20), V6-7 (17/20), and V4 (16/20) followed closely behind, whereas V8 assigned 15/20, and V9 was only able to identify two species (2/20) (Table 2).

**TABLE 2:** Observed species rRNA gene abundance denoted as percent of total.

Species	Expected	V2	V3	V4	V6-7	V8	V9
*Acinetobacter baumannii*	5.26	6.23	6.06	6.82	0.11	10.90	46.15
*Actinomyces odontolyticus*	1.75	1.75	0.27	1.51	0.97	1.54	0.00
*Bacillus cereus*	10.52	5.20	10.29	6.97	6.99	1.21	0.00
*Bacteroides vulgatus*	6.14	13.11	8.19	12.48	18.57	0.00	0.00
*Bifidobacterium adolescentis*	4.39	5.37	0.00	4.22	0.00	5.85	0.00
*Clostridium beijerinckii*	12.28	0.00	0.00	0.00	19.94	0.12	0.00
*Deinococcus radiodurans*	6.14	9.88	0.00	1.47	3.29	6.31	0.00
*Enterococcus faecalis*	3.51	1.97	3.41	0.00	1.53	3.88	0.00
*Escherichia coli*	6.14	5.64	6.52	4.77	8.29	0.00	53.73
*Helicobacter pylori*	1.75	5.70	2.24	2.90	2.68	0.51	0.00
*Lactobacillus gasseri*	5.26	4.45	7.21	0.00	0.00	0.00	0.00
*Neisseria meningitidis*	3.51	5.14	4.13	6.05	0.00	9.07	0.00
*Porphyromonas gingivalis*	3.51	2.85	5.83	7.77	8.83	0.00	0.00
*Propionibacterium acnes*	3.51	1.35	0.27	0.17	1.97	6.04	0.00
*Pseudomonas aeruginosa*	3.51	5.20	5.58	2.79	2.00	7.62	0.00
*Rhodobacter sphaeroides*	2.63	3.75	4.10	3.83	2.87	12.88	0.00
*Staphylococcus aureus*	5.26	3.19	5.44	5.71	3.00	2.08	0.00
*Staphylococcus epidermidis*	4.39	3.44	5.40	0.00	2.49	0.23	0.00
*Streptococcus agalactiae*	6.14	1.89	6.31	5.46	3.06	0.00	0.00
*Streptococcus mutans*	4.39	0.87	5.47	4.81	2.09	8.03	0.00
Total Species Identified	20	19	17	16	17	15	2

**FIGURE 5:**
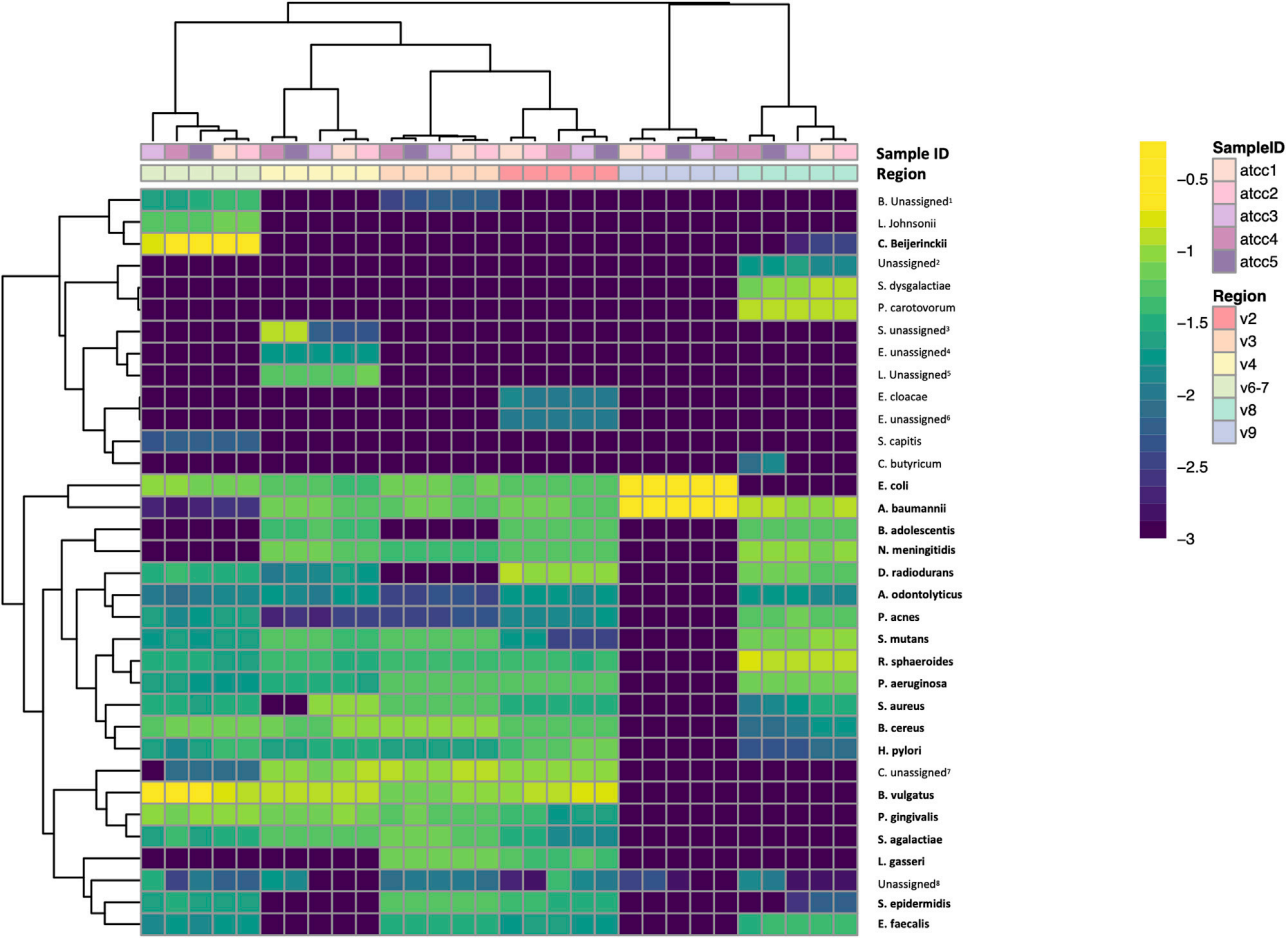
Species-level clustered heatmap of ATCC 20 Strain Even Mix. Bolded taxa are those present in the 20 strain mix. *Bifidobacterium* unassigned^1^, *Enterobacteriaceae* unassigned^2^, *Staphylococcus* unassigned^3^, *Enterococcus* unassigned^4^, *Lactobacillus* unassigned^5^, *Enterobacter* unassigned^6^, *Clostridium sensu stricto 1* unassigned^7^, Unassigned at every taxonomic level^8^.

Lastly, we performed a clustered heatmap analysis at the species level. The resulting heatmap demonstrated that technical replicates of the mock community sequences cluster by hypervariable region (Figure 5). The heatmap visually emphasizes the difference in taxonomic identification in V8 and particularly V9 compared to the other regions. It also highlights misclassifications and which regions were only able to classify taxa to the genus level. Interestingly, the heatmap highlights a few misclassifications or false negatives that occurred in only a subset of the replicates. For example, *Staphylococcus aureus* was classified as *Staphyloccoccus* unassigned in replicates four and five. The OTU tables for these samples indicate that the sequence was truncated prematurely in replicates four and five, indicating the differences in classification here arise from library preparation or sequencing errors rather than downstream data analysis.

### Taxonomic Classification of Human Gut Microbiome Culture Collection

Since there appeared to be differing abilities of classification of bacterial species by hypervariable region in our ATCC data set, we next determined if this was the case for a larger pool of bacteria. We plotted out the taxonomic classification results from our *in silico* database validation to visualize whether sensitivity and specificity was region specific (Supplementary Figure S1). The sensitivity and mis-classification rates varied with respect to particular species and hypervariable regions. For example, *Bifidobacterium longum* is 100% misassigned when using sequences from V4, but no other region. This region likewise has 0% sensitivity for *B. longum*. Alternatively, *Bifidobacterium bifidum* has high specificity across all hypervariable regions, implying that sensitivity and specificity of taxonomic classification may be increased by using data from multiple hypervariable regions.

### Clinical Samples

We next sequenced and analyzed a set of six patient samples in order to demonstrate the use of a generalized linear model (GLM) in an illustrative clinical sample set, incorporating information from multiple hypervariable regions. Hypervariable regions V2, V3, V4, and V6-7 were included in the GLM, while data from the V8 and V9 regions were excluded due to their demonstrated poor performance in identifying species in the mock community (Figures 2–5). Samples consisted of duplicate rectal swabs from three participants. DNA was extracted immediately after collection from one rectal swab sample chosen at random from each patient (fresh) and the other sample was frozen at -80°C prior to DNA isolation (frozen). Libraries were prepared in tandem, and all samples were sequenced on the same sequencing run. Sequencing results were processed as outlined above (Figure 1).

**FIGURE 6:**
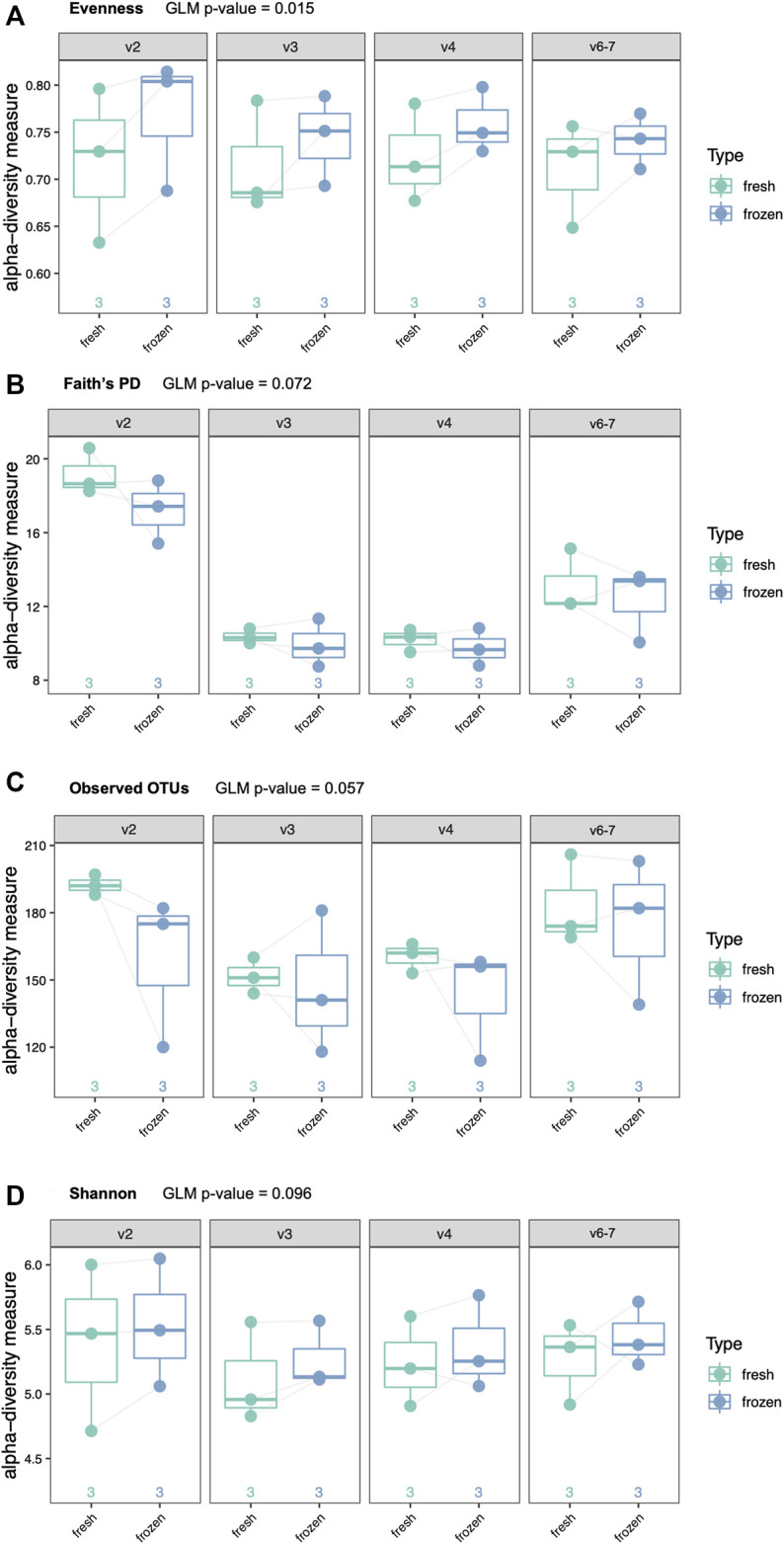
Alpha diversity analyses of six clinical samples by type (fresh or frozen) and hypervariable region. Each patient provided two swabs, one of which was frozen prior to DNA extraction. **(A)** Evenness (*p* = 0.015), **(B)** Faith’s phylogenetic diversity (*p* = 0.072), **(C)** Observed Operational Taxonomic Units (OTUs) (*p* = 0.067), **(D)** Shannon diversity (*p* = 0.096).

We performed the same four alpha diversity metrics for the clinical cohort as for the mock community samples (evenness, Shannon diversity, observed OTUs, and Faith’s phylogenetic diversity). There were no significant differences in alpha diversity between fresh and frozen samples by Shannon diversity, Faith’s phylogenetic diversity or observed OTUs when using a GLM (Figure 6). Evenness was slightly increased in frozen samples across all hypervariable regions (adjusted GLM *p* = 0.015).

**FIGURE 7:**
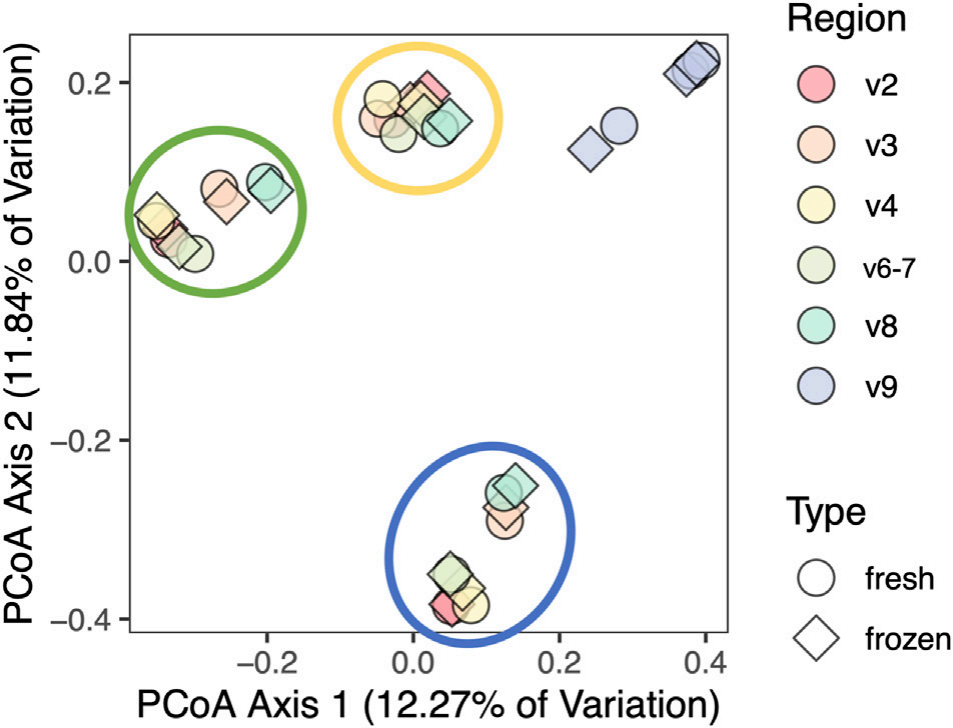
Principal coordinates analysis of clinical cohort using Canberra distance matrix. Samples and regions from the same person are circled, excluding V9. Results cluster by individual and by V9 region (not circled), but not by fresh versus frozen status.

We aggregated taxonomic results and used them to create Bray-Curtis, Jaccard, Canberra, Euclidean, Gower, and Kulczynski distance matrices in order to perform combined beta diversity analysis across all hypervariable regions. As demonstrated by the Canberra PCoA plot in Figure 7, most variation in beta diversity was due to different individuals and V9 sequences. PERMANOVA analysis of results from each individual hypervariable region demonstrated that total composition does not differ by fresh versus frozen status after adjusting for individual person and region-to-region variation (Supplementary File S4).

**FIGURE 8:**
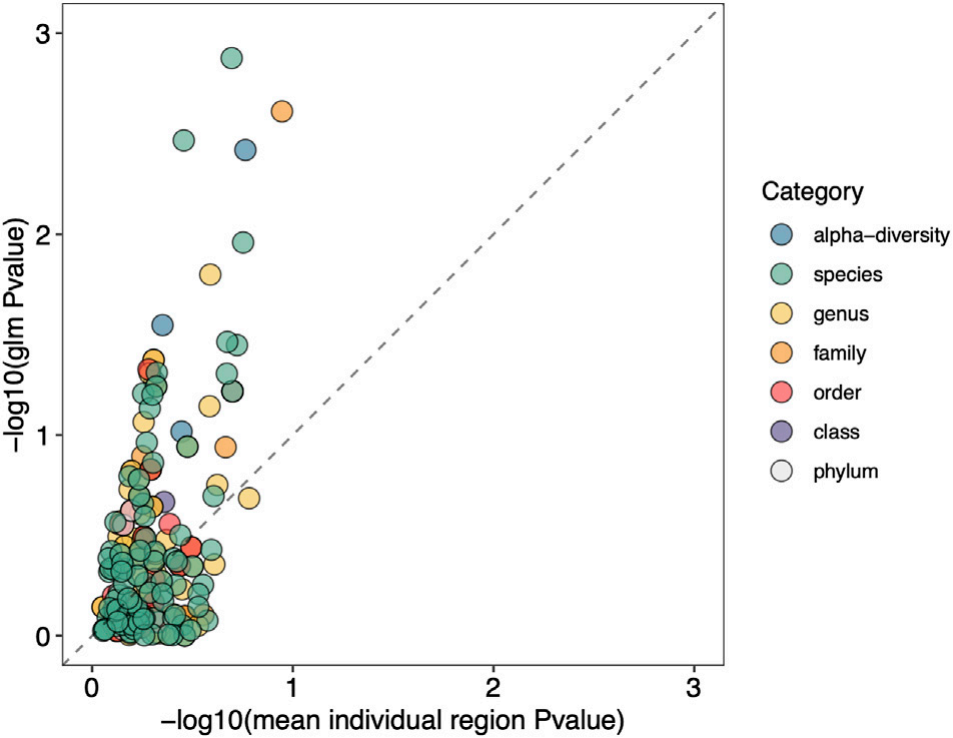
Using a GLM shows enrichment of taxonomic classification sensitivity. GLM p-values for specific taxa are plotted on the *y*-axis, and the mean p-value across all hypervariable regions for the same taxa are plotted on the *x*-axis. *p*-values are log-transformed and multiplied by -1 so that more significant p-values are higher in value. The dashed line indicates where the p-values resulting from the GLM and from individual regions are equal. Enrichment above the dashed line indicates the GLM approach is more sensitive compared to analyzing individual regions.

We next show that using a GLM that incorporates information from multiple variable regions increases the ability to detect significant differences between groups. This is demonstrated in Figure 8, where we plot the average p-value for each specific taxon across all hypervariable regions against the p-value obtained for the same taxon when using a GLM. Due to small sample size, we opted to use unadjusted p-values. There is an enrichment of significant p-values when using the GLM as seen by the shift upwards above the dashed line, indicating an increase in sensitivity compared to analyzing individual hypervariable regions.

**FIGURE 9:**
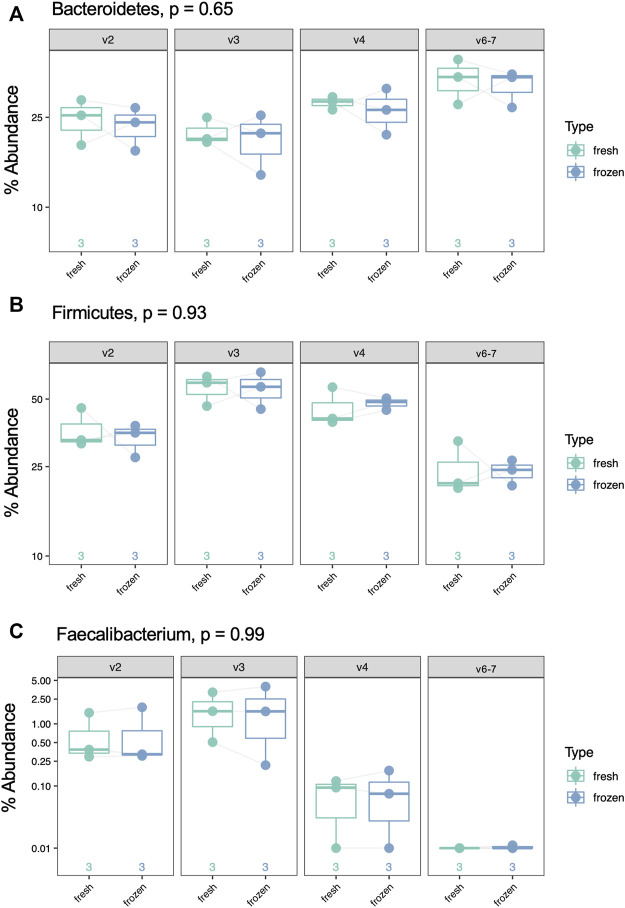
Percent abundance of *Bacteroidetes*, *Firmicutes*, and *Faecalibacterium* by sample type (fresh *vs* frozen) and hypervariable region. *p*-value was calculated with a log-transformed GLM and is false discovery rate-adjusted. **(A)** Bacteriodetes, *p* = 0.65, **(B)** Firmicutes, *p* = 0.93, **(C)** Faecalibacterium, *p* = 0.99.

**FIGURE 10:**
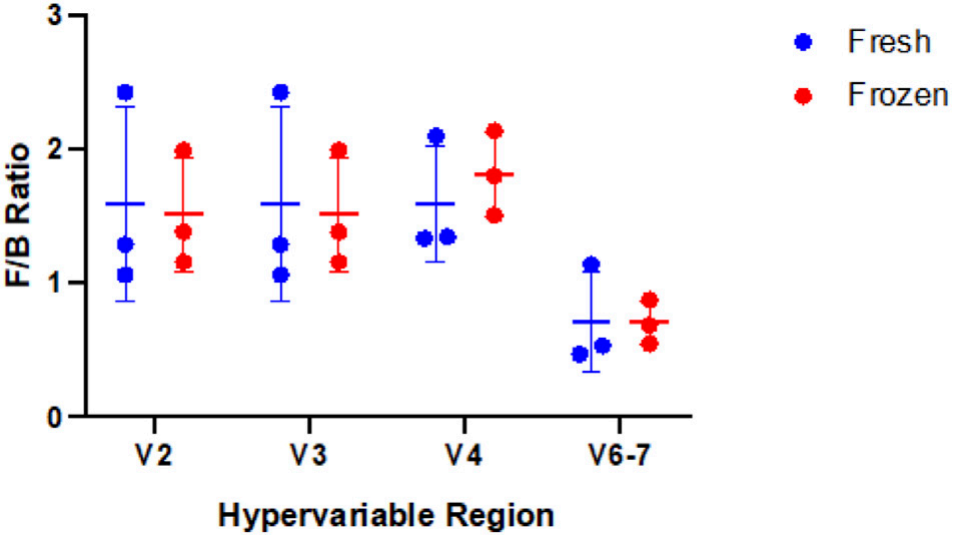
Comparison of *Firmicutes* to *Bacteroidetes* (F/B) ratio in fresh versus frozen samples by hypervariable region. No significant difference was observed between fresh and frozen samples for the hypervariable regions (V2 p-value = 0.87, V3 p-value = 0.87, V4 p-value = 0.51, V6-7 p-value = 0.97).

Using our GLM, we systematically compared abundance of taxa between fresh and frozen samples at multiple levels (phylum, class, order, family, genus, species). As an example, we chose to examine levels of *Firmicutes*, *Bacteroidetes*, and *Faecalibacterium* due to previous reports of differential abundance in fresh verses frozen samples (Bahl et al., 2012; Fouhy et al., 2015). Our results showed no significant differences between these taxa (Figure 9) or *Firmicutes* to *Bacteroidetes* ratios (Figure 10). While no concrete conclusions can be made from this data due to small sample size, we demonstrate the utility of the GLM using clinical samples.

## Discussion

16 S rRNA sequencing is cost effective, requires relatively low DNA input, and has a number of highly curated reference databases and open-source analysis platforms, making it a common tool for microbiome researchers. PCR amplification using primers that target conserved regions of the 16 S rRNA gene and amplify across hypervariable regions allows amplification of DNA across a widespread taxonomic spectrum and provides unique sequences that can be used for taxonomic classification at higher levels (e.g., family, genus, and species level). Next generation sequencing strategies are often limited to sequencing across only one or at most two of the nine hypervariable regions. The Ion 16 S™ Metagenomics Kit provides the opportunity to prepare libraries containing sequences from seven of the nine hypervariable regions (V2, V3, V4, V6-7, V8, and V9). However, the Ion Reporter analysis pipeline available to Ion 16 S™ Metagenomics Kit users does not allow users to incorporate their own study metadata into analyses and does not allow users to export usable data for downstream analyses, necessitating the development of open-resource analysis tools for data produced from the Ion 16 S™ Metagenomics Kit.

Herein, we report results from sequencing a mock microbial community using the Ion 16 S™ Metagenomics Kit and comparing results from different hypervariable regions. Using a cohort of clinical samples, we demonstrate that taxonomic classification is enhanced by using a generalized linear multivariate model (GLM) that incorporates sequencing data from multiple hypervariable regions.

We first prepared and sequenced five technical replicates of DNA from a twenty strain mock microbial community, and then assessed alpha diversity (evenness, Shannon diversity, observed OTUs, and Faith’s phylogenetic diversity) among different hypervariable regions. Even with our limited mock community dataset, we observed hypervariable region-based differences in alpha diversity. Most notably, taxa identified with V9 primers had significantly decreased alpha diversity compared to all other regions across all metrics. V8 results likewise had significantly decreased Shannon Diversity and Faith’s PD, suggesting that V8 and V9 are falsely underrepresenting the diversity of the samples.

We performed six different beta diversity metrics (Bray-Curtis, Jaccard, Canberra, Euclidean, Gower, and Kulczynski) to evaluate differences between hypervariable regions. Distance matrices used in beta diversity analyses are generated from OTU tables, however the OTUs identified were not consistent among hypervariable regions. Therefore, in order to compare results between hypervariable regions, we assembled distance matrices using taxonomic results. PCoA analyses demonstrated clustering primarily by hypervariable regions V2, V3, V4, and V6-7. Hypervariable regions V8 and V9 clustered separately from the other regions, again demonstrating the poor performance of amplicon sequencing of these regions in assessing the constituents of the mock community sample.

Consistent with previous reports (Claesson et al., 2010; Cai et al., 2013; Tremblay et al., 2015; Barb et al., 2016), we found that the taxonomic classification results from the mock community samples varied by hypervariable region. Primers targeting the V2, V3, and V6-7 regions identified nearly all the species present in the mock community (19/20, 17/20, and 17/20 respectively), V4 identified 16/20 species, V8 identified 15/20 species, and V9 identified only two (2/20) (Figure 3; Table 2). Generally, those regions which identified more species present in the mock community also had more evenly distributed observed taxa (i.e., there were no extreme over- or underestimated taxa which skewed the remaining percent abundances, such as in the case of V9).

Errors and biases that contribute to artifacts in PCR-based microbiome studies include sequence artifacts (formation of chimeras or heteroduplexes, or polymerase errors), PCR bias (differing amplification efficiencies of different templates), or biases in the analysis pipeline (poorly discriminatory sequences) (Acinas et al., 2005). Of all OTUs assigned to the V9 region, only two OTUs made up 99.78% of total V9 reads. Therefore, we deduce that the lack of diversity in the region is likely most related to PCR bias. Since V9 lacks sensitivity for many species, we opted to leave this region out of the generalized linear model we used on the clinical samples. V8 also tended to be less sensitive compared to V2, V3, V4, and V6-7, and contributed to variation in the data according to PCoA plots. Therefore, V8 was excluded from further analyses as well. Notably, primer sequences for this kit are not available, and having access to primer sequences in this instance would aid in delving further into why V8 and V9 provided so little information. For others attempting to incorporate a GLM into their analysis, we would recommend against using data from V8 and V9. One must also take into account whether specific regions have increased or decreased sensitivity for specific taxa of interest when considering which regions to include in your GLM.

Researchers can circumvent the issue of choosing only one hypervariable region to analyze by sequencing multiple hypervariable regions in tandem. Since the sensitivity of each hypervariable region for identifying bacterial taxa varies, combining the results from multiple hypervariable regions for analyses may be misleading. Fuks et al. developed Short MUltiple Regions Framework (SMURF), which combines sequences from multiple PCR amplicons in order to provide one overall set of taxonomic profiling results (Fuks et al., 2018). However, this method is computationally intensive and requires proprietary software. Therefore, to utilize information from multiple hypervariable regions at once and to strengthen confidence in the taxonomic abundance results, we incorporated a generalized linear model (GLM) into alpha diversity and taxonomic abundance analyses.

We demonstrated use of the GLM *via* analysis of a clinical cohort, where each participant donated two rectal swab samples, one of which was processed fresh and the other one frozen prior to DNA extraction. Alpha diversity analysis revealed increased evenness in frozen samples compared to fresh samples. This trend was visualized in results from each individual hypervariable region and was strengthened in the GLM. There was no difference in Shannon’s diversity, observed OTUs, and Faith’s phylogenetic diversity between fresh and frozen samples which suggests that freezing samples may not affect the ability to detect taxa, but it might alter the detectable abundance of certain taxa. Beta diversity analysis demonstrated clustering of samples by person irrespective of fresh versus frozen status or hypervariable region, with the exception of V9. PERMANOVA analysis confirmed that most of the variation in composition was due to individuals as opposed to storage type. An important limitation of our beta diversity analysis is that in order to compare results from all hypervariable regions in the same analysis, we had to use taxonomic classification as opposed to OTUs. This limits our beta diversity analysis to using only those reads that were assigned taxonomy.

By utilizing a GLM with sequences from our clinical samples, sensitivity to changes between groups was enriched compared to using only one hypervariable region. *p*-values for specific differences in taxa between fresh and frozen samples became significant when utilizing sequences from multiple hypervariable regions, while one region was not powerful enough to detect these differences as observed in Figure 8.

Finally, based on the findings above, we compared taxonomic abundance at multiple levels between fresh and frozen samples using a GLM. We found no taxa at any level had significantly different abundance. This is unsurprising based on our small sample size, the fact that alpha and beta diversity were minimally different between sample type, and the fact that other studies show limited differences between fresh verses frozen samples (Bahl et al., 2012; Fouhy et al., 2015). However, *Faecalibacterium* results highlight the important point that not all regions are able to identify a taxon of interest: V6-7 fails to map any reads to this taxon despite its presence in the sample. Thus, even though the true composition of a clinical sample may be unknown, examining redundant data from multiple hypervariable regions may help elucidate the true microbial makeup of the sample, with the caveat that none of the hypervariable regions included vary too significantly from the others to prevent skewing the data.

In conclusion, we propose a method to overcome the issues of analyzing multiple amplicons covering multiple hypervariable regions at once. While this protocol is tailored towards analyzing data generated from the Ion Torrent platform, the approach of sequencing multiple hypervariable regions and analyzing data in parallel could be applied towards Illumina sequencing data, as well. As more tools to analyze more of the 16 S rRNA gene at once become available, it is critical for the microbiome bioinformatics community to come to a consensus as to the proper way to analyze this type of data in order to maintain data quality, and to be able to compare results across different publications.

## Data Availability

The datasets presented in this study can be found in online repositories. The names of the repository/repositories and accession number(s) can be found below: https://www.ncbi.nlm.nih.gov/, PRJNA738491 http://github.com/Sfanos-Lab-Microbiome-Projects/it-workflow/.

## Supplementary Material

Supplementary Figure S1 Taxonomic sensitivity and mis-classification rates of human gut microbiome culture collection by hypervariable region.

Supplementary Table S1 List of contaminants. Supplementary File S1. *In silico* taxonomic validation results.

Supplementary File S2 Mock community alpha diversity statistics.

Supplementary File S3 Mock community filtered percent abundance.

Supplementary File S4 PERMANOVA analysis of fresh *vs* frozen clinical samples.
