# Supplementary figures and images for: Incorporation of Data From Multiple Hypervariable Regions when Analyzing Bacterial 16S rRNA Gene Sequencing Data
Source: Front Genet. 2022 Mar 31;13:799615. doi: 10.3389/fgene.2022.799615 (PMC9009396; doi:10.3389/fgene.2022.799615)

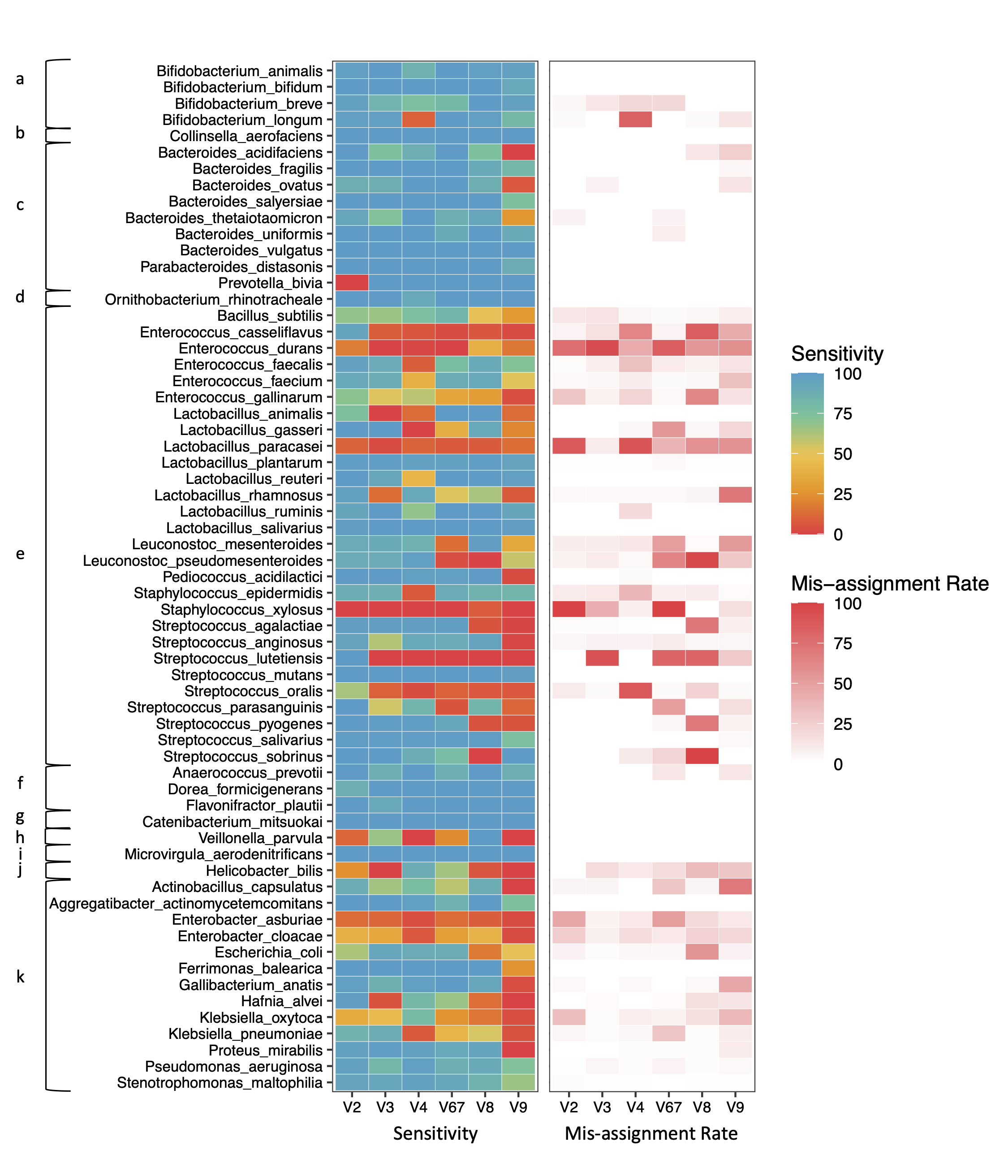

Supplement: Supplementary file 1 [file Image1.TIFF]
